# Incidence profile of four major cancers among migrants in Australia, 2005–2014

**DOI:** 10.1007/s00432-023-04764-5

**Published:** 2023-04-19

**Authors:** Xue Qin Yu, Marianne Weber, David Smith, Louiza Velentzis, Erich V. Kliewer, Michael David, Eleonora Feletto

**Affiliations:** 1 The Daffodil Centre, The University of Sydney, a joint venture with Cancer Council NSW, Kings Cross, PO Box 572, Sydney, NSW 1340 Australia; 2 School of Public Health and Preventive Medicine, Monash University, Melbourne, Australia; 3 Cancer Control Research, BC Cancer, Vancouver, Canada; 4 School of Medicine and Dentistry, Griffith University, Gold Coast, Australia

**Keywords:** Cancer, Migrants, Epidemiology, Australia

## Abstract

**Purpose:**

To compare the incidence profile of four major cancers in Australia by place of birth.

**Methods:**

In this retrospective population-based cohort study, the analysis included 548,851 residents diagnosed with primary colorectum, lung, female breast, or prostate cancer during 2005–2014. Incidence rate ratio (IRR) and 95% confidence intervals (CI) were calculated for migrant groups relative to Australian-born.

**Results:**

Compared with Australian-born residents, most migrant groups had significantly lower incidence rates for cancers of the colorectum, breast and prostate. The lowest rates of colorectal cancer were among males born in Central America (IRR = 0.46, 95% CI 0.29–0.74) and females born in Central Asia (IRR = 0.38, 95% CI 0.23–0.64). Males born in North-East Asia had the lowest rates of prostate cancer (IRR = 0.40, 95% CI 0.38–0.43) and females born in Central Asia had the lowest rates of breast cancer (IRR = 0.55, 95% CI 0.43–0.70). For lung cancer, several migrant groups had higher rates than Australian-born residents, with the highest rates among those from Melanesia (males IRR = 1.39, 95% CI 1.10–1.76; females IRR = 1.40, 95% CI 1.10–1.78).

**Conclusions:**

This study describes cancer patterns among Australian migrants, which are potentially helpful in understanding the etiology of these cancers and guiding the implementation of culturally sensitive and safe prevention measures. The lower incidence rates observed for most migrant groups may be maintained with continued emphasis on supporting communities to minimize modifiable risk factors such as smoking and alcohol consumption and participation in organized cancer screening programmes. Additionally, culturally sensitive tobacco control measures should be targeted to migrant communities with high lung cancer incidence rates.

**Supplementary Information:**

The online version contains supplementary material available at 10.1007/s00432-023-04764-5.

## Introduction

Cancer is the leading cause of disease burden in Australia, with cancers of the breast, prostate, colorectum, and lung among the commonest, constituting 45% of all cancers combined in 2018 (Australian Institute of Health and Welfare 2021). The incidence rates of these cancers varied markedly globally, from more than threefold variation in breast cancer, ninefold in colorectal cancer, to over 13-fold in prostate cancer, with Australia/New Zealand among the regions with the highest rates (Sung et al. 2021). Lung cancer, the second commonest cancer globally in 2020, had an over 20-fold variation in incidence between regions (Sung et al. 2021). The underlying drivers of these global patterns are multi-faceted and are likely to be associated with variation in the prevalence of exposures, access to cancer screening and detection technologies, and genetic factors (Australian Institute of Health and Welfare 2021; Sung et al. 2021).

Australia is home to approximately 7.6 million migrants, accounting for 30% of its population (Australian Bureau of Statistics 2021). Over the past several decades, Australia has evolved into a nation of people from over 190 countries and 300 different ancestries (Australian Bureau of Statistics 2021). Due to its diverse population and comprehensive cancer registration (Bray et al. 2017), Australia is well suited for studies of cancer in migrant populations. Prior Australian data showed that migrants to Australia generally have lower rates of many common cancers than native-born Australians (McCredie et al. 1994; McCredie et al. 1990a, b; McMichael and Bonett 1981; McMichael et al. 1980). However, these studies reported on relatively large, heterogenous countries of birth (e.g., Asia being classified as one group) (McCredie et al. 1990b), were state-specific or are now dated (McCredie et al. 1990b, 1994; McMichael and Bonett 1981). A contemporary analysis of the incidence of major cancers by migrant groups is warranted given the changing sociodemographic profile of migrants over time (Parliamentary Library 2018), and changing rates worldwide in these cancers over time (Sung et al. 2021). These findings potentially help understand the etiology of these cancers and guide the implementation of culturally sensitive and safe prevention measures. Therefore, this study aimed to compare the incidence of four major cancers among Australian migrants to the native-born Australians.

## Methods

### Study population

The number of colorectal, lung, female breast, and prostate cancer cases diagnosed during 2005–2014 by country of birth (COB) and region of birth, sex, 5 year age groups and year of diagnosis were obtained from the Australian Cancer Database (Australian Institute of Health and Welfare 2019), which compiles national data from the eight state and territory population-based cancer registries. By law, all cancers diagnosed in Australia must be reported to the relevant jurisdictional register.

The corresponding population data, estimated resident population (ERP), were obtained from the Australian Bureau of Statistics (Australian Bureau of Statistics 2019). The ERP by COB for each between-census year were estimated by interpolation, using administrative data relating to births, deaths, and overseas migration together with routinely collected information on COB for Australian residents during quinquennial population censuses.

Migrants were defined based on their COB, consistent with the most widely used definition in migrant studies (Arnold et al. 2010). Australian cancer registries routinely collect this information, generally from hospital inpatient records, supplemented with death records if the patient has died. We reported 12 COBs with relatively large populations individually (in the top ten countries for the overseas-born population in either the 2006 or 2011 Census, plus Ireland), and 17 regions with additional countries included in their relevant regions (Table 1), based on the Standard Australian Classification of Countries (SACC) (Australian Bureau of Statistics 2016). Cases for whom the cancer registry had missing COB data were excluded from the analysis.

**Table 1:** List of countries included in each region of birth group

Region of birth (SACC code)	Individual countries^†^
Melanesia (1300)	New Caledonia, Papua New Guinea, Solomon Islands, Vanuatu
Polynesia (1500)	Cook Island, Fiji, French Polynesia, Niue, Samoa, Samoa American, Tokelau, Tonga, Tuvalu, Wallis & Futuna, Pitcairn Islands, Polynesia (excludes Hawaii) not elsewhere classified
Western Europe (2300)	Austria, Belgium, France, Germany, Liechtenstein, Luxembourg, Monaco, Netherlands, Switzerland
Northern Europe (2400)	Denmark, Faroe Islands, Finland, Greenland, Iceland, Norway, Sweden, Aland Islands
Southern Europe (3100)	Andorra, Gibraltar, Holy See, Italy, Malta, Portugal, San Marino, Spain
South Eastern Europe (3200)	Albania, Bosnia & Herzegovina, Bulgaria, Croatia, Cyprus, the former Yugoslav Republic of Macedonia, Greece, Moldova, Romania, Slovenia, Montenegro, Serbia, Kosovo
Eastern Europe (3300)	Belarus, Czech Republic, Estonia, Hungary, Latvia, Lithuania, Poland, Russian Federation, Slovakia, Ukraine
North Africa (4100)	Algeria, Egypt, Libya, Morocco, Sudan, Tunisia, Western Sahara, Spanish North Africa, South Sudan
Middle East (4200)	Bahrain, Gaza Strip & West Bank, Iran, Iraq, Israel, Jordan, Kuwait, Lebanon, Oman, Qatar, Saudi Arabia, Syria, Turkey, United Arab Emirates, Yemen
South-East Asia (5000)	Myanmar, Cambodia, Laos, Thailand, Vietnam, Brunei Darussalam, Indonesia, Malaysia, Philippines, Singapore, Timor-Leste
North-East Asia (6000)	China, Hong Kong (SAR of China), Macau (SAR of China), Mongolia, Taiwan, Japan, North Korea, South Korea
Southern Asia (7100)	Bangladesh, Bhutan, India, Maldives, Nepal, Pakistan, Sri Lanka
Central Asia (7200)	Afghanistan, Armenia, Azerbaijan, Georgia, Kazakhstan, Kyrgyzstan, Tajikistan, Turkmenistan, Uzbekistan
Northern America (8100)	Bermuda, Canada, St Pierre and Miquelon, United States of America
South America (8200)	Argentina, Bolivia, Brazil, Chile, Colombia, Ecuador, Falkland Islands, French Guiana, Guyana, Paraguay, Peru, Suriname, Uruguay, Venezuela
Central America (8300)	Belize, Costa Rica, El Salvador, Guatemala, Honduras, Mexico, Nicaragua, Panama
Southern & East Africa (9200)	Angola, Botswana, Burundi, Comoros, Djibouti, Eritrea, Ethiopia, Kenya, Lesotho, Madagascar, Malawi, Mauritius, Mayotte, Mozambique, Namibia, Reunion, Rwanda, St Helena, Seychelles, Somalia, South Africa, Swaziland, Tanzania, Uganda, Zambia, Zimbabwe, Southern & Eater Africa, Africa not elsewhere classified

^†^Based on the Standard Australian Classification of Countries (SACC) 2016 (March 2017 release)

*SAR* special administrative region

### Statistical analyses

Three regions (Micronesia, Caribbean, and Central and West Africa) were excluded from these analyses as their expected number of new cases was < 50 per cancer type/sex/region combinations (Yu et al. 2022). Data were analyzed by sex because of the anticipated differences in the prevalence of exposures and variations in screening or testing participation.

Age-standardized incidence rates (per 100,000) were calculated for each migrant group by cancer type using the world standard population (Bray et al. 2017). Incidence rate ratios (IRRs) and 95% confidence intervals (CI) were calculated for each migrant group relative to the Australian-born population, using a negative binomial regression model adjusted for age group, year of diagnosis, and the log of the population as an offset variable. All analyses were performed using SAS version 9.4 (SAS Institute Inc) and the figures were produced using R 4.0.5 (R Core Team, 2021).

## Results

Over half a million (548,851) Australian residents diagnosed with one of the four selected cancers during 2005–2014 were included in the analysis. Overall, 6.7% of the records had missing COB data, ranging from 2.0% for lung cancer to 10.4% for prostate cancer. The highest proportion of cancer cases that were Australian-born were those with prostate cancer (71%), followed by colorectal and breast cancer (69%), and lung cancer (64%).

**Fig. 1:**
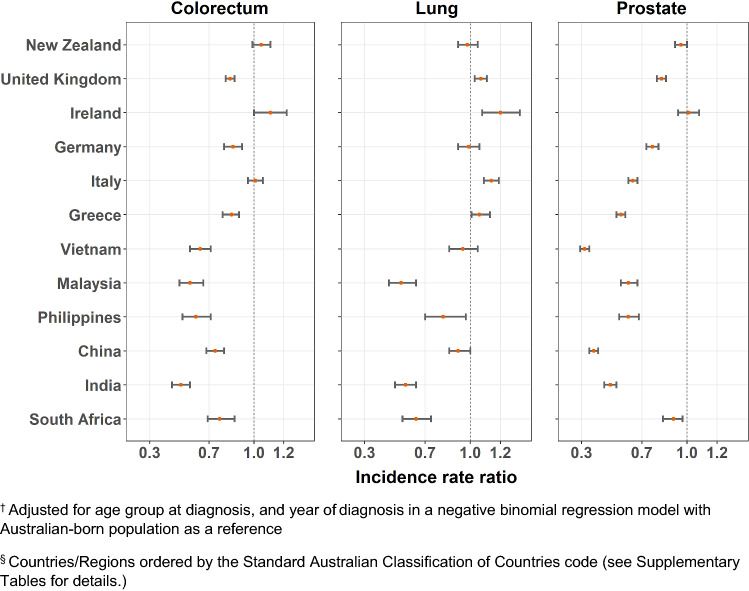
Adjusted incidence rate ratio^†^ by country of birth^§^ relative to the Australian-born residents by cancer type, males

**Fig. 2:**
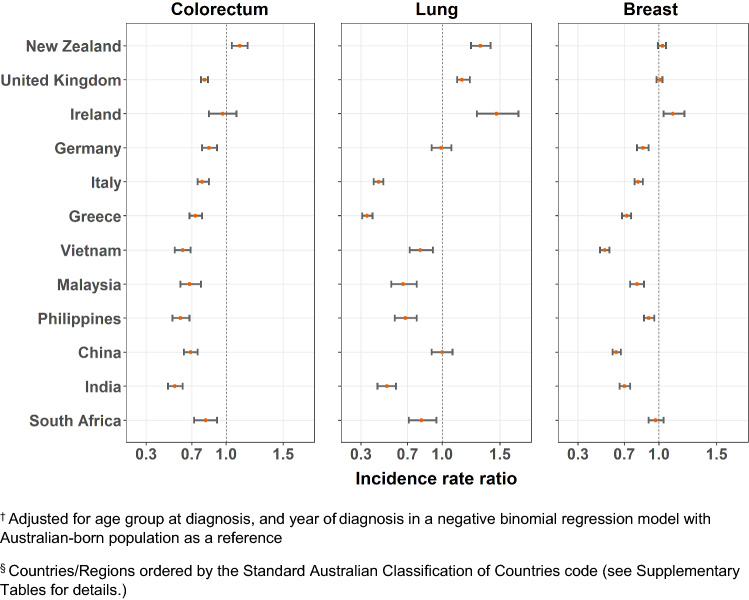
Adjusted incidence rate ratio^†^ by country of birth^§^ relative to the Australian-born residents by cancer type, females

**Fig. 3:**
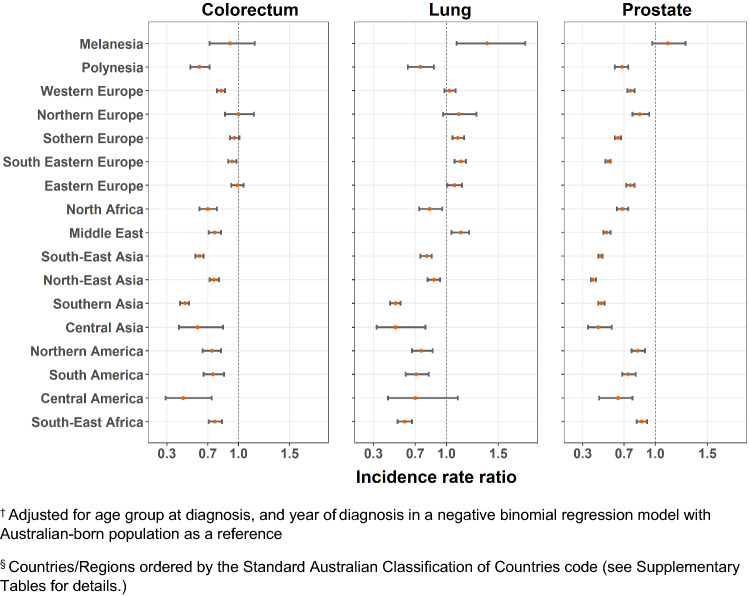
Adjusted incidence rate ratio^†^ by region of birth^§^ relative to the Australian-born residents by cancer type, males

**Fig. 4:**
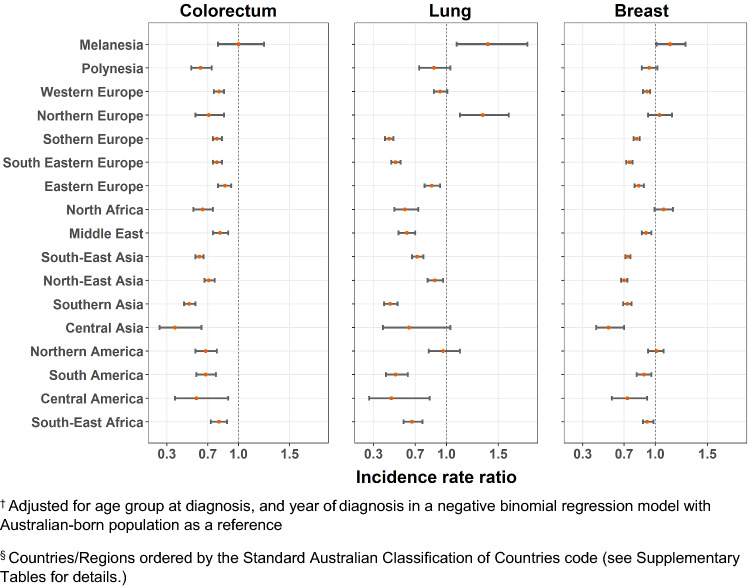
Adjusted incidence rate ratio^†^ by region of birth^§^ relative to the Australian-born residents by cancer type, females. ^†^Adjusted for age group at diagnosis, and year of diagnosis in a negative binomial regression model with Australian-born population as a reference. ^§^Countries/Regions ordered by the Standard Australian Classification of Countries code (see Supplementary Tables for details.)

Age-standardized incidence rates and adjusted IRRs are presented in Supplementary Tables 1 and 2, with significant variation observed for each cancer type by migrant groups (*p* < 0.0001). The adjusted IRRs were plotted in Figs. 1, 2, 3 and 4. Incidence rates were generally lower in most migrant groups for colorectal (for males and females), breast and prostate cancer, except in migrants from English-speaking countries (UK, Ireland, New Zealand, South Africa) for whom rates were relatively higher or closer to the Australian-born rates. For lung cancer, several migrant groups had higher rates than the Australian-born.

### Colorectal cancer

Most migrant groups had lower rates of colorectal cancer than the Australian-born population, with the lowest rates observed in migrants from India (IRR M 0.51–F 0.55), and Southern Asia (M 0.48–F 0.52). Only males from Ireland (1.11, 95% CI 1.00–1.22) and females from New Zealand (1.12, 95% CI 1.05–1.19) had higher rates than the Australian-born population.

### Lung cancer

In males with lung cancer, migrants from five of 17 regions (Melanesia, Southern Europe, South Eastern Europe, Eastern Europe and the Middle East) had significantly higher incidence rates than their Australian-born counterparts, with the highest rates among migrants from Melanesia (IRR = 1.39, 95% CI 1.10–1.76). Migrants from four countries had significantly higher rates (UK, Ireland, Italy, and Greece), with Irish migrants having the highest rate (1.20, 95% CI 1.08–1.33). In contrast, rates were lower among migrants from four countries and nine regions. Migrants from Southern Asia (0.51, 95% CI 0.46–0.56) and Central Asia (0.51, 95% CI 0.33–0.80) had the lowest rates.

In females, two regions and three countries had higher rates of lung cancer than their Australian-born counterparts, with the highest rates among migrants from Ireland (IRR = 1.47, 95% CI 1.30–1.66) and the Melanesian region (1.40, 95% CI 1.10–1.78). Rates were lower for migrants from seven countries and 11 regions, with the lowest rate being for Greek migrants (0.35, 95% CI 0.31–0.40).

### Breast cancer

Females from 12 of 17 regions had significantly lower incidence rates than Australian-born females. Lower rates were predominantly observed among females from Central Asia (IRR = 0.55, 95% CI 0.43–0.70), with Vietnamese females having the lowest rates (0.53, 95% CI 0.49–0.57). Irish and Melanesian migrants had higher rates than Australian-born females (1.12, 95% CI 1.04–1.22; 1.14, 95% CI 1.01–1.29, respectively).

### Prostate cancer

Except for three migrant groups (New Zealand, Ireland, and the Melanesia region), which had similar incidence rates to Australian-born males, all migrant groups had lower incidence rates than Australian-born males. Two migrant groups with the lowest rates were those from Vietnam (IRR = 0.32, 95% CI 0.29–0.35), China (0.38, 95% CI 0.35–0.41).

## Discussion

Between 2005 and 2014, almost all Australian migrant groups had significantly lower incidence rates for cancers of the colorectum, breast and prostate than their Australian-born counterparts. For lung cancer, rates were lower than Australian-born residents for many migrant groups, but males born in Melanesia and various European regions and females born in Melanesia and Northern Europe had higher rates than their Australian-born counterparts. Thus, further research focusing on the differences in behavior, attitudes, risk factors and health-related characteristics between these migrant groups could provide more insights into the etiology of these cancers. Studies examining migrant groups with low cancer rates may particularly provide insights on how to reduce the incidence in the Australian-born population.

Breast, lung and colorectal cancer are associated with modifiable risk factors (e.g., tobacco smoking, reproductive factors, excess body weight and diet) that vary in prevalence globally (Anand et al. 2008). However, socio-cultural factors and screening or testing behaviors may also be associated with the observed variation in incidence rates (Arnold et al. 2010). Overall, exposures in the COB and acculturation—a process whereby migrants adopt their host country’s exposures (e.g., changes to diet, reproductive practices, and environmental exposures)—may be important in driving patterns of incidence in Australian migrant communities. However, we were unable to account for these factors in this study due to limitations with available data.

Consistent with previous Australian studies, lower breast cancer rates were observed for most migrant groups than Australian-born females. Although differences in genetic factors cannot be ruled out, evidence suggests that other known risk factors (e.g., alcohol consumption, menopausal hormone therapy use) contribute to the higher rates for Australian-born population (Gathani et al. 2014). Lower incidence for females from non-English-speaking countries may suggest a lower prevalence of risk factors among them, e.g., lower alcohol consumption among Chinese, Vietnamese, Italian, Greek and Middle Eastern women (Duncan 2010), alongside a higher prevalence of factors associated with lower breast cancer risk, e.g., lower body mass index (BMI) (Menigoz et al. 2016), and higher rates to initiate and maintain breastfeeding among female migrants (Dennis et al. 2019), one of the strongest protective factors. The lower screening participation rate for many non-English speaking migrants than English speakers (Australian Institute of Health and Welfare 2018) may also contribute to their lower incidence. The similar or higher rates for females born in English-speaking countries suggest that risk factors and possible screening participation may be similar across these countries and Australia (Abbasi-Shavazi and McDonald 2000). Interventions targeting under-screened migrant groups, co-designed with these communities to ensure culturally appropriate delivery would help increase participation in BreastScreen. Closing the equity gap in accessing screening could also be achieved by broadening the source used to identify and invite women to join BreastScreen; currently, the electoral roll is used as the source, which does not capture permanent residents covered by Medicare.

As other Australian studies have found (McCredie et al. 1994; McCredie et al. 1990a, b; McMichael and Bonett 1981; McMichael et al. 1980), most migrant groups had a lower incidence of colorectal cancer than the Australian-born. The high rate in Australian-born residents mirrors global patterns (Sung et al. 2021), and may be partially attributed to the high prevalence of established risk factors, including physical inactivity and excess body weight, heavy alcohol consumption, red/processed meat consumption, and low consumption of dietary fiber (Johnson et al. 2013). The lower odds of colorectal cancer screening (up to 40% lower than locally born participants) among people from non-English speaking backgrounds (Australian Institute of Health and Welfare 2018) may also have contributed to their lower incidence. This may be due to delayed acculturation, as there is evidence of increased screening rates with longer duration of residence in Australia (Weber et al. 2009). Additionally, screening programs may not exist in their home country, with limited awareness or acceptance of screening practices and stigma associated with screening.

A higher incidence of lung cancer was observed among several migrant groups. Higher rates for UK/Irish migrants were consistent with previous studies (McCredie et al. 1990b; McMichael and Bonett 1981), while our findings for the other migrant groups contrast to prior study results indicating lower rates for Middle Eastern (McCredie et al. 1994) and Southern European migrants (including Italy and Greece) (McCredie et al. 1990a). These patterns likely reflect differences in smoking patterns among these migrant groups. Prior Australian studies found that males born in parts of Europe, and the Middle East (Department of Health NSW 2012; Siahpush and Borland 2001; Weber et al. 2011), and females born in New Zealand and UK/Ireland were more likely to smoke (Siahpush and Borland 2001; Weber et al. 2011) than their Australian-born counterparts. As the stages of the tobacco epidemic (i.e., the comparative levels of smoking prevalence and smoking-attributed mortality) have varied globally over time, and given the 20–30 year lag between tobacco exposure and lung cancer incidence (Drope and Schluger 2018), it is of interest to continue monitoring lung cancer rates across migrant groups. The lung cancer treatment and detection landscape is evolving rapidly in Australia, with a potential lung cancer screening program for individuals with a history of heavy smoking on the horizon (Australian Institute of Health and Welfare 2021). Key to its success will be engaging those with the highest risk, including the migrant groups identified here. Further, lung cancer among individuals without a smoking history will also be important to monitor, especially among Asian-born females who have disproportionately higher rates (Cheng et al. 2022).

Prostate cancer rates were lower among most migrant groups than Australian-born males, particularly for males from Asian countries, with rates 50–60% lower in migrants from Southern and North–East Asia. Evidence of well-established risk factors is limited. While age, family history, and specific genetic mutations are shown to increase risk, associations between modifiable factors e.g., diet, BMI and smoking are less clear (World Cancer Research Fund & American Institute for Cancer Research 2018). Internationally, African American men and Caribbean men of African ancestry have some of the highest risk of prostate cancer. The proportion of migrants from these ethnic groups is relatively small in Australia, thus our data do not allow for meaningful comparison. Much of the global variation in incidence (Sung et al. 2021) appears to be related to the prevalence of prostate cancer testing (Zhou et al. 2016). The patterns observed among Australian migrants may be associated with the prevalence of prostate-specific antigen (PSA) testing, however, studies from New South Wales have shown somewhat inconsistent evidence. Some earlier studies suggested that urban-dwelling East Asian males and Chinese-born males had lower PSA testing rates (30 and 50% less, respectively) than Australian-born males based on self-reported data (Litchfield et al. 2012; Weber et al. 2009, 2014), while a more recent analysis based on Medicare claims showed few differences in PSA testing across major migrant groups (Nair-Shalliker et al. 2018). This variation may suggest increased adoption of testing behaviors in migrant groups or issues surrounding health literacy and accuracy of self-reporting of testing behaviors by migrants from non-English speaking backgrounds. Current Australian guidelines regarding testing and treatment for prostate cancer recommend informed decision-making regarding PSA testing for asymptomatic men aged 50–69 (Prostate Cancer Foundation of Australia 2016). Migrants for whom English is not a first language may be less likely to make well-informed decisions about prostate cancer testing, and future development of culturally appropriate resources may be a priority.

The use of recent national cancer incidence data is a strength of this study, providing contemporary insights into the cancer profile of migrants to Australia. Of note, incidence data are superior to mortality data in etiology studies as mortality is influenced by treatment-related factors, which may also vary between population subgroups (McMichael and Bonett 1981). Additionally, the cohort size was sufficiently large to report cancer profiles for many smaller migrant groups, including those from Melanesia, which had higher lung and female breast cancer rates. These cancer rates may provide estimates for their countries of origin due to minimal local cancer registry data. This suggests that future studies should disaggregate migrant groups to reflect the diversity because of their heterogeneous incidence patterns. Also, we used the annual ERP data by COB to provide more accurate rate estimates than the quinquennial census population data used in many previous Australian studies (McCredie et al. 1990a, b; McMichael et al. 1980).

The exclusion of 10.4% of the records with unknown COB for prostate cancer was a limitation and may have affected these results. Many men with low-risk prostate cancer may choose active surveillance without needing hospital care (McIntosh et al. 2022) which is the primary source of the COB data recorded in the cancer registries. Another limitation is that data on the age of migration or length of residence in Australia were not available for analysis, thus we were unable to investigate the impact of acculturation on cancer rates.

The incidence patterns observed suggest that the lower rates for most migrant groups may be maintained with continued emphasis on healthy lifestyle behaviors and uptake of organized screening in culturally safe and sensitive ways. For lung cancer, this is particularly important for tobacco control measures which could be best targeted to those migrant communities identified in this study with higher incidence rates.

## Supplementary Information

Below is the link to the electronic supplementary material.

Supplementary file1 (DOCX 22 KB)

Supplementary file2 (DOCX 22 KB)

## Data Availability

This study involves the analysis of aggregated data from the Australian Cancer Database, managed by the Australian Institute of Health and Welfare (AIHW). Data may be available from the corresponding author with approval from the data custodian in each state and territory of Australia.
