# Supplementary material for: Incidence profile of four major cancers among migrants in Australia, 2005–2014
Source: J Cancer Res Clin Oncol. 2023 Apr 19;149(11):8317–25. doi: 10.1007/s00432-023-04764-5 (PMC10374701; doi:10.1007/s00432-023-04764-5)
Supplement: Supplementary file 1 — Supplementary file1 (DOCX 22 KB) [file 432_2023_4764_MOESM1_ESM.docx]

**Supplementary Table** 1 Age-standardised incidence rates and adjusted incidence rate ratios^†^ for colorectal, lung and prostate cancer among males by place of birth, 2005-2014

| Place of birth (SACC code) | **Colorectum** | | | | **Lung** | | | | **Prostate** | | | |
| --- | --- | --- | --- | --- | --- | --- | --- | --- | --- | --- | --- | --- |
|  | Cases (n) | Rate* | IRRs | 95% CI | Cases (n) | Rate* | IRRs | 95% CI | Cases (n) | Rate* | IRRs | 95% CI |
| **Country of birth** | | | | | | | | | | | | |
| Australian-born (1101) | 50347 | 43.8 | 1.00 |  | 38293 | 32.6 | 1.00 |  | 124858 | 113.9 | 1.00 |  |
| New Zealand (1201) | 1583 | 46.0 | 1.05 | (0.99-1.11) | 1072 | 31.9 | 0.98 | (0.92-1.05) | 3762 | 109.5 | 0.96 | (0.92-1.00) |
| United Kingdom (2100) | 7160 | 36.8 | 0.84 | (0.81-0.87) | 7559 | 35.2 | 1.07 | (1.03-1.11) | 18139 | 95.3 | 0.83 | (0.80-0.86) |
| Ireland (2201) | 437 | 48.6 | 1.11 | (1.00-1.22) | 371 | 35.9 | 1.20 | (1.08-1.33) | 1010 | 119.4 | 1.01 | (0.94-1.08) |
| Germany (2304) | 944 | 37.4 | 0.86 | (0.80-0.92) | 865 | 31.2 | 0.99 | (0.92-1.06) | 2269 | 88.3 | 0.77 | (0.73-0.81) |
| Italy (3104) | 2826 | 43.1 | 1.01 | (0.96-1.06) | 2646 | 35.0 | 1.14 | (1.09-1.19) | 4436 | 74.0 | 0.64 | (0.61-0.67) |
| Greece (3207) | 1284 | 38.3 | 0.85 | (0.79-0.90) | 1322 | 38.1 | 1.06 | (1.01-1.13) | 2185 | 64.4 | 0.56 | (0.53-0.59) |
| Vietnam (5105) | 369 | 26.0 | 0.64 | (0.57-0.71) | 392 | 29.3 | 0.95 | (0.86-1.05) | 452 | 37.0 | 0.32 | (0.29-0.35) |
| Malaysia (5203) | 201 | 24.8 | 0.57 | (0.50-0.66) | 139 | 17.1 | 0.54 | (0.46-0.64) | 570 | 70.4 | 0.61 | (0.56-0.67) |
| Philippines (5204) | 152 | 26.9 | 0.61 | (0.52-0.71) | 140 | 28.8 | 0.82 | (0.70-0.97) | 376 | 73.0 | 0.61 | (0.55-0.68) |
| China (6101) | 723 | 32.3 | 0.74 | (0.68-0.80) | 694 | 29.2 | 0.92 | (0.86-1.00) | 915 | 41.8 | 0.38 | (0.35-0.41) |
| India (7103) | 321 | 22.6 | 0.51 | (0.45-0.57) | 263 | 17.6 | 0.57 | (0.50-0.64) | 749 | 55.1 | 0.49 | (0.45-0.53) |
| South Africa (9225) | 320 | 33.5 | 0.77 | (0.69-0.87) | 192 | 21.1 | 0.64 | (0.55-0.74) | 954 | 102.5 | 0.91 | (0.84-0.97) |
| **Region of birth** | | | | | | | | | | | | |
| Melanesia (1300) | 67 | 37.8 | 0.92 | (0.72-1.16) | 72 | 45.5 | 1.39 | (1.10-1.76) | 194 | 126.8 | 1.12 | (0.97-1.29) |
| Polynesia (1500) | 172 | 25.6 | 0.62 | (0.53-0.72) | 148 | 23.8 | 0.75 | (0.63-0.88) | 477 | 80.9 | 0.68 | (0.61-0.74) |
| Western Europe (2300) | 2103 | 36.8 | 0.83 | (0.79-0.87) | 2171 | 33.6 | 1.03 | (0.98-1.09) | 5110 | 88.6 | 0.76 | (0.73-0.80) |
| Northern Europe (2400) | 218 | 42.9 | 1.00 | (0.87-1.15) | 196 | 35.1 | 1.12 | (0.97-1.29) | 488 | 101.5 | 0.85 | (0.78-0.94) |
| Southern Europe (3100) | 3465 | 41.3 | 0.96 | (0.92-1.01) | 3415 | 36.1 | 1.11 | (1.06-1.17) | 5798 | 74.4 | 0.64 | (0.61-0.67) |
| South Eastern Europe (3200) | 3171 | 41.5 | 0.94 | (0.90-0.98) | 3100 | 39.3 | 1.14 | (1.08-1.19) | 4881 | 62.5 | 0.55 | (0.52-0.57) |
| Eastern Europe (3300) | 1309 | 42.7 | 0.99 | (0.93-1.05) | 1207 | 36.8 | 1.08 | (1.01-1.15) | 2429 | 89.2 | 0.76 | (0.72-0.80) |
| North Africa (4100) | 269 | 31.2 | 0.70 | (0.62-0.79) | 259 | 26.8 | 0.84 | (0.74-0.96) | 677 | 76.6 | 0.68 | (0.63-0.74) |
| Middle East (4200) | 734 | 33.4 | 0.77 | (0.71-0.83) | 814 | 37.5 | 1.14 | (1.05-1.22) | 1317 | 61.0 | 0.53 | (0.50-0.57) |
| South-East Asia (5000) | 1072 | 26.3 | 0.62 | (0.58-0.66) | 1023 | 25.9 | 0.81 | (0.75-0.86) | 2070 | 55.1 | 0.47 | (0.45-0.49) |
| North-East Asia (6000) | 1083 | 33.1 | 0.76 | (0.72-0.81) | 956 | 28.2 | 0.88 | (0.82-0.94) | 1423 | 45.9 | 0.40 | (0.38-0.43) |
| Southern Asia (7100) | 497 | 21.2 | 0.48 | (0.43-0.52) | 395 | 16.4 | 0.51 | (0.46-0.56) | 1238 | 55.4 | 0.48 | (0.45-0.51) |
| Central Asia (7200) | 32 | 27.5 | 0.60 | (0.42-0.85) | 19 | 16.9 | 0.51 | (0.33-0.80) | 57 | 50.4 | 0.45 | (0.35-0.58) |
| Northern America (8100) | 288 | 32.3 | 0.74 | (0.65-0.83) | 221 | 25.8 | 0.76 | (0.67-0.87) | 846 | 95.4 | 0.83 | (0.77-0.90) |
| South America (8200) | 241 | 33.0 | 0.75 | (0.66-0.86) | 170 | 23.3 | 0.71 | (0.61-0.83) | 636 | 85.4 | 0.74 | (0.68-0.81) |
| Central America (8300) | 17 | 16.5 | 0.46 | (0.29-0.74) | 18 | 24.5 | 0.70 | (0.44-1.11) | 57 | 70.6 | 0.64 | (0.46-0.78) |
| Southern-East Africa (9200) | 559 | 33.5 | 0.77 | (0.71-0.84) | 315 | 20.0 | 0.60 | (0.53-0.67) | 1587 | 97.7 | 0.87 | (0.82-0.92) |

* Age-standardised incidence rates per 100,000.

^†^ Adjusted for age group at diagnosis, and year of diagnosis in a negative binomial regression model with Australian-born population as a reference.

Abbreviations: SACC - the Standard Australian Classification of Countries; IRR - incidence rate ratio; CI – confidence intervals.
