# Supplementary material for: Incidence profile of four major cancers among migrants in Australia, 2005–2014
Source: J Cancer Res Clin Oncol. 2023 Apr 19;149(11):8317–25. doi: 10.1007/s00432-023-04764-5 (PMC10374701; doi:10.1007/s00432-023-04764-5)
Supplement: Supplementary file 2 — Supplementary file2 (DOCX 22 KB) [file 432_2023_4764_MOESM2_ESM.docx]

**Supplementary Table** 2 Age-standardised incidence rates and adjusted incidence rate ratios^†^ for colorectal, lung and breast cancer among females by place of birth, 2005-2014

| Place of birth  (SACC code) | **Colorectum** | | | | **Lung** | | | | **Breast** | | | |
| --- | --- | --- | --- | --- | --- | --- | --- | --- | --- | --- | --- | --- |
|  | Cases (n) | Rate* | IRRs | 95% CI | Cases (n) | Rate* | IRRs | 95% CI | Cases (n) | Rate* | IRRs | 95% CI |
| **Country of birth** | | | | | | | | | | | | |
| Australian-born (1101) | 44174 | 31.5 | 1.00 |  | 27806 | 21.1 | 1.00 |  | 91560 | 83.8 | 1.00 |  |
| New Zealand (1201) | 1228 | 35.4 | 1.12 | (1.05-1.19) | 963 | 28.0 | 1.33 | (1.25-1.42) | 3191 | 86.0 | 1.03 | (0.99-1.06) |
| United Kingdom (2100) | 5186 | 25.3 | 0.81 | (0.78-0.84) | 5338 | 25.0 | 1.19 | (1.13-1.24) | 12415 | 83.6 | 1.01 | (0.98-1.03) |
| Ireland (2201) | 268 | 29.4 | 0.97 | (0.85-1.09) | 264 | 29.0 | 1.47 | (1.30-1.66) | 609 | 95.5 | 1.12 | (1.04-1.22) |
| Germany (2304) | 752 | 28.0 | 0.85 | (0.79-0.92) | 580 | 19.9 | 0.99 | (0.91-1.08) | 1312 | 71.0 | 0.86 | (0.81-0.91) |
| Italy (3104) | 1528 | 26.8 | 0.79 | (0.75-0.85) | 567 | 9.0 | 0.45 | (0.41-0.49) | 2353 | 71.6 | 0.82 | (0.79-0.86) |
| Greece (3207) | 799 | 24.0 | 0.73 | (0.68-0.79) | 261 | 8.1 | 0.35 | (0.31-0.40) | 1292 | 63.3 | 0.72 | (0.68-0.76) |
| Vietnam (5105) | 310 | 18.6 | 0.62 | (0.55-0.69) | 263 | 16.5 | 0.81 | (0.72-0.92) | 760 | 42.6 | 0.53 | (0.49-0.57) |
| Malaysia (5203) | 213 | 21.5 | 0.68 | (0.60-0.78) | 139 | 13.9 | 0.66 | (0.56-0.78) | 712 | 67.8 | 0.81 | (0.75-0.87) |
| Philippines (5204) | 280 | 17.3 | 0.60 | (0.53-0.68) | 210 | 14.7 | 0.68 | (0.59-0.78) | 1366 | 74.4 | 0.91 | (0.87-0.96) |
| China (6101) | 597 | 21.2 | 0.69 | (0.63-0.75) | 551 | 20.2 | 1.00 | (0.91-1.09) | 1321 | 52.9 | 0.63 | (0.60-0.67) |
| India (7103) | 267 | 17.3 | 0.55 | (0.49-0.62) | 159 | 10.3 | 0.52 | (0.44-0.60) | 811 | 58.8 | 0.70 | (0.66-0.75) |
| South Africa (9225) | 277 | 24.6 | 0.82 | (0.72-0.92) | 183 | 17.1 | 0.82 | (0.71-0.95) | 885 | 81.8 | 0.97 | (0.91-1.04) |
| **Region of birth** | | | | | | | | | | | | |
| Melanesia (1300) | 76 | 28.7 | 1.00 | (0.80-1.25) | 69 | 28.4 | 1.40 | (1.10-1.78) | 266 | 97.2 | 1.14 | (1.01-1.29) |
| Polynesia (1500) | 150 | 19.1 | 0.63 | (0.54-0.74) | 139 | 18.9 | 0.88 | (0.74-1.04) | 671 | 78.2 | 0.94 | (0.87-1.02) |
| Western Europe (2300) | 1498 | 26.3 | 0.81 | (0.76-0.86) | 1228 | 19.8 | 0.94 | (0.88-1.01) | 2983 | 76.2 | 0.92 | (0.88-0.95) |
| Northern Europe (2400) | 102 | 22.3 | 0.71 | (0.58-0.86) | 134 | 28.8 | 1.35 | (1.13-1.60) | 325 | 86.9 | 1.04 | (0.93-1.16) |
| Southern Europe (3100) | 1908 | 26.1 | 0.79 | (0.75-0.84) | 766 | 9.7 | 0.45 | (0.41-0.49) | 3172 | 71.8 | 0.82 | (0.79-0.85) |
| South-east Europe (3200) | 1835 | 25.7 | 0.79 | (0.75-0.84) | 838 | 11.9 | 0.51 | (0.47-0.56) | 3285 | 63.1 | 0.75 | (0.72-0.78) |
| Eastern Europe (3300) | 1001 | 27.4 | 0.87 | (0.80-0.93) | 678 | 17.7 | 0.86 | (0.79-0.94) | 1489 | 71.4 | 0.84 | (0.80-0.89) |
| North Africa (4100) | 175 | 20.8 | 0.65 | (0.56-0.75) | 112 | 12.3 | 0.60 | (0.50-0.73) | 569 | 92.2 | 1.08 | (0.99-1.17) |
| Middle East (4200) | 510 | 25.9 | 0.82 | (0.75-0.90) | 260 | 13.0 | 0.62 | (0.54-0.70) | 1522 | 76.7 | 0.91 | (0.87-0.96) |
| South-east Asia (5000) | 1125 | 18.9 | 0.62 | (0.58-0.66) | 867 | 14.9 | 0.72 | (0.67-0.78) | 3919 | 60.3 | 0.73 | (0.71-0.76) |
| North-east Asia (6000) | 904 | 22.0 | 0.71 | (0.67-0.77) | 748 | 18.4 | 0.89 | (0.82-0.97) | 2372 | 57.8 | 0.70 | (0.67-0.73) |
| Southern Asia (7100) | 410 | 16.4 | 0.52 | (0.47-0.58) | 240 | 9.5 | 0.46 | (0.40-0.53) | 1428 | 61.0 | 0.73 | (0.69-0.77) |
| Central Asia (7200) | 15 | 11.4 | 0.38 | (0.23-0.64) | 16 | 13.8 | 0.64 | (0.39-1.04) | 67 | 44.1 | 0.55 | (0.43-0.70) |
| Northern America (8100) | 178 | 21.1 | 0.68 | (0.58-0.79) | 170 | 20.8 | 0.97 | (0.83-1.13) | 761 | 85.4 | 1.01 | (0.93-1.08) |
| South America (8200) | 188 | 21.4 | 0.68 | (0.59-0.78) | 97 | 10.7 | 0.51 | (0.42-0.63) | 642 | 74.0 | 0.89 | (0.82-0.96) |
| Central America (8300) | 21 | 16.0 | 0.59 | (0.38-0.90) | 11 | 10.3 | 0.47 | (0.26-0.84) | 74 | 59.1 | 0.73 | (0.58-0.92) |
| Southern-East Africa (9200) | 460 | 24.6 | 0.81 | (0.73-0.89) | 258 | 14.2 | 0.67 | (0.59-0.77) | 1445 | 77.5 | 0.92 | (0.88-0.98) |

* Age-standardised incidence rates per 100,000.

^†^ Adjusted for age group at diagnosis, and year of diagnosis in a negative binomial regression model with Australian-born population as a reference.

Abbreviations: SACC - the Standard Australian Classification of Countries; IRR - incidence rate ratio; CI – confidence intervals.
